# Immune Checkpoint Inhibitors and Male Fertility: Should Fertility Preservation Options Be Considered before Treatment?

**DOI:** 10.3390/cancers16061176

**Published:** 2024-03-17

**Authors:** Elissavet Ntemou, Emily Delgouffe, Ellen Goossens

**Affiliations:** Genetics, Reproduction and Development (GRAD) Research Group, Biology of the Testis (BITE) Team, Vrije Universiteit Brussel (VUB), 1090 Brussels, Belgium; emily.delgouffe@vub.be (E.D.); ellen.goossens@vub.be (E.G.)

**Keywords:** immune checkpoint inhibitors, CTLA-4, PD-1, PD-L1, spermatogenesis, testis, male fertility

## Abstract

**Simple Summary:**

Recently, a new type of cancer treatment called immune checkpoint inhibitors (ICIs) has become an option for many cancer patients, including children. While these treatments are effective against different types of cancer, they can lead to immune-related side effects impacting different organs. However, knowledge about the effect of ICIs on testicular function and male fertility is limited. There is a possibility that ICI treatment directly or indirectly affects testicular function and sperm production. This review looks at the available evidence on how ICIs, especially those targeting cytotoxic T-lymphocyte-associated protein 4 (CTLA-4), programmed death protein 1 (PD-1) and programmed death-ligand 1 (PD-L1), may disrupt sperm production. It also emphasizes the need for further investigations and encourages discussions about associated risks and fertility-preservation options between clinicians and patients.

**Abstract:**

In recent years, immune checkpoint inhibitors (ICIs) have become a viable option for many cancer patients, including specific subgroups of pediatric patients. Despite their efficiency in treating different types of cancer, ICIs are responsible for a number of immune-related adverse events, including inflammatory toxicities, that can affect several organs. However, our knowledge of the impact of ICIs on the testis and male fertility is limited. It is possible that ICI treatment affects testicular function and spermatogenesis either directly or indirectly (or both). Treatment with ICIs may cause increased inflammation and immune cell infiltration within the seminiferous tubules of the testis, disturbing spermatogenesis or testosterone deficiency (primary hypogonadism). Additionally, the interference of ICIs with the hypothalamic–pituitary–gonadal axis may alter testosterone production, affecting testicular function (secondary hypogonadism) and spermatogenesis. This review provides an overview of the available evidence on the potential association between ICIs and the disruption of spermatogenesis, with special focus on ICIs targeting cytotoxic T-lymphocyte-associated protein 4 (CTLA-4), programmed death protein 1 (PD-1) and programmed death-ligand 1 (PD-L1). Moreover, it highlights the need for further investigations and encourages the discussion of associated risks and fertility-preservation considerations between clinicians and patients.

## 1. Introduction

Over the last decade, a transformative shift in the landscape of cancer treatment has been witnessed, moving away from traditional cytotoxic therapies to more specific and targeted treatments. Immune checkpoint inhibitors (ICIs) represent a novel class of immunotherapeutic agents, designed to enhance the patient’s immune response against cancer cells by targeting immune checkpoint pathways. Most clinically approved ICIs are designed to target the specific immune checkpoint molecules cytotoxic T-lymphocyte-associated protein 1 (CTLA-4), programmed death protein 1 (PD-1), and programmed death-ligand 1 (PD-L1), which typically act to suppress immune responses [1]. The impact of the discovery of cancer therapy that inhibits negative immune regulation was highlighted when the 2018 Nobel Prize in Physiology or Medicine was awarded to James Allison and Tasuku Honjo for their pioneering contribution to the field [2].

While the medical community celebrates the successes of ICIs in achieving durable responses and improved survival rates for various cancers, there is a growing concern about their side effects. ICIs have been associated with a spectrum of inflammatory toxicities, known as immune-related adverse effects (irAEs). These irAEs can affect various organ systems such as the gastrointestinal tract, liver, lungs, pituitary and thyroid glands, skin, heart, and nervous system [3]. Despite these observations, the understanding of the potential impact of ICIs on testicular function and male fertility remains limited. Importantly, this concern not only worries adult men but also pediatric patients for whom ICI therapy is used. Unlike conventional cancer treatments, such as chemotherapy and radiation, which are known to directly affect the testicular cells and often prompt recommendations for fertility preservation [4], the unique mechanisms of action of ICIs introduce a new layer of complexity in understanding their effects on the male reproductive system.

This comprehensive review aims to summarize the existing knowledge and highlight the areas that require further investigation regarding the potential male reproductive risks associated with ICI therapy. Special attention will be given to the immunoregulation of the testis and its unique immune-privileged status, along with the physiological and hormonal changes observed during different developmental stages. The overarching goal of this overview is to address the potential need for fertility preservation before ICI treatment and encourage discussions on this matter between clinicians and patients, thereby enhancing the comprehensive care provided to male individuals undergoing ICI therapy.

## 2. Immune Checkpoint Inhibitors

### 2.1. Mechanism of Action

The intricate regulation of T-cell activation and functionality in cell-mediated tumor immunity requires a balance between stimulatory and inhibitory signals. A major step in this process involves the presentation of tumor-associated antigens to T-cell receptors, giving specificity to the immune response [5]. Co-regulatory signals, mediated by ligand–receptor interactions with both agonistic and antagonistic effects, contribute to the dynamics of T-cell activation and differentiation.

Immune checkpoints negatively regulate T-cell outcomes. CTLA-4 is a surface receptor prominently expressed by activated T-cells, and its role is to suppress further activation. Additionally, CTLA-4 is involved in the activation of regulatory T-cells, whose function is to suppress immune responses and maintain immune homeostasis [6]. PD-1 is a surface receptor expressed by various immune cells, including B cells and tumor-infiltrating lymphocytes, natural killer cells, and some myeloid cell populations, but it is mainly expressed by all activated T-cells upon their entry into the periphery and initiation of the effector phase [7]. Its role is to regulate effector T-cell function in peripheral tissues during various physiological responses, including acute and chronic infection, cancer, and autoimmunity, and in immune homeostasis [8]. PD-L1 is commonly expressed in many tumor cells [9]. The binding of PD-1 to its ligand PD-L1 suppresses further activation, diminishes inflammation, and prevents autoimmunity [10]. Notably, this interaction between PD-1 and PD-L1 is used advantageously by tumor cells, which upregulate PD-L1 expression to avoid immune responses [11].

ICIs act by targeting specific molecules and pathways and regulate the immune system by activating and enhancing the body’s tumor-specific immune response. In addition, they contribute to the formation of memory T-cells for prolonged protection against cancer recurrence. More specifically, CTLA-4 inhibitors enhance T-cell activation and promote an immune response against cancer cells by blocking the downregulation of immune responses, while PD-1/PD-L1 inhibitors allow T-cells to effectively attack cancer cells by blocking inhibitory interactions [12]. Notably, the blockade of immune checkpoints may exert an influence on T-regulatory cells, potentially initiating autoimmune and inflammatory irAEs [13].

### 2.2. Available Therapies

The application of ICI therapy took a significant step when the FDA approved ipilimumab (anti-CTLA-4) in 2011. This approval set the stage for a series of others, including pembrolizumab (anti-PD-1) and atezolizumab (anti-PD-L1), in the following years [14]. As of 23 January 2024, the US clinical trials database [15] reports a substantial 739 ongoing clinical trials for anti-PD-1, 361 for anti-PD-L1, and 199 for anti-CTLA-4. Beyond these established targets, the landscape of ICI therapy is evolving, with investigations into other novel targets such as BTLA, VISTA, TIM-3, LAG-3, and CD47, emphasizing the continued exploration and investment in this field [16]. The initial scope of ICI therapy in specific recurrent or advanced malignancies has evolved significantly. Their success stimulated exploration in neoadjuvant (pre-surgery) and adjuvant (post-surgery) settings, underlining their growing relevance in the management of early-stage diseases [17]. ICIs are already used to treat a wide range of cancer types, as they have been effectively deployed against multiple solid tumors, with approvals for several cancer indications and tumors with mismatch repair mutations. Their usage is anticipated to continue growing in the future [18]. A comprehensive overview of FDA-approved ICIs for adult cancer patients, along with their indications, is presented in Table 1 [19].

Moreover, nowadays, the use of ICIs extends beyond the adult population, with emerging FDA approvals for pediatric patients. (Table 2) [19,20]. At the moment, the majority of ICIs in clinical trials involving pediatric patients include children 12 years of age and older, with the only exception being trials for atezolizumab (anti-PD-L1), which is intended for children from 2 years of age and older. However, the increasing clinical use of ICIs also brings new challenges, as more patients (including children) may achieve durable remission and will have to deal with the long-term side effects associated with their treatment.

## 3. Immunoregulation of the Testis

The mammalian testis possesses a unique immunoregulatory environment that is essential for testicular function [21]. As an immune-privileged site, the testis prevents an immune response against the immunogenic germ cells, while the local innate immunity is essential in preventing microbial infections within the testicular environment. The tight regulation of this environment is imperative to maintain immune homeostasis for normal spermatogenesis [22]. The blood–testis barrier (BTB), together with the distinctive immune cell repertoire and the combined release of immunostimulative and immunosuppressive factors by Leydig cells, Sertoli cells, and peritubular cells, collectively contributes to the maintenance of a tolerogenic environment within the testis [23]. Disturbance in testicular immune homeostasis may result in autoimmune infertility and increase susceptibility to testicular infections, leading to orchitis [23].

### 3.1. The Blood–Testis Barrier

The BTB is established through various junctions, such as the tight junction, basal ectoplasmic specialization, gap junction, and desmosome-like junction, between adjacent Sertoli cells and divides the seminiferous epithelium into basal and adluminal compartments [24]. Thus, with the adluminal compartment being secluded from vascular and lymphatic vessels, the selective passage of essential nutritional molecules and growth factors to post-meiotic cells is facilitated by the BTB. Most importantly, this isolation establishes an environment where post-meiotic cells are shielded against immunological attacks and the development of anti-sperm antibodies. This protective mechanism effectively supports spermatogenesis and prevents infertility [25].

### 3.2. Immune Cell Repertoire in the Testis

Immune cells have a critical role in maintaining testicular homeostasis by mitigating the inflammatory response and supporting normal physiological functions. Several immune cell types are present in the testis including macrophages, dendritic cells, mast cells and T-cells contributing towards maintaining the tolerogenic immune state [26].

Macrophages are the most abundant and heterogeneous immune-cell population found in the testis and exist in close physical and functional association with Leydig cells [27]. Testicular macrophages demonstrate limited ability to produce proinflammatory cytokines, in contrast to macrophages found in other tissues. They produce interleukin (IL)-10 and exhibit an immunosuppressive phenotype [28,29]. Their role includes the suppression of T-cell proliferation and activation, facilitating the differentiation of naive T-cells into immunosuppressive regulatory T-cells. Moreover, they serve testis-specific functions essential for maintaining normal homeostasis, including guiding testis embryonic development, supporting steroidogenesis, and promoting spermatogenesis [30].

Dendritic cells are also found in the interstitium. These cells are “professional” antigen-presenting cells and a cellular component of the adaptive immune system. Under physiological conditions, immature dendritic cells support the immune privilege status of the testis and suppress the activation of T-cells, while under inflammatory conditions, mature dendritic cells proliferate and stimulate effector T-cell expansion [31]. In infertile patients with chronic inflammation, both macrophages and dendritic cell numbers are increased, and their functions are compromised [32].

Mast cells play diverse roles in innate immunity, tissue homeostasis, and remodeling, as well as adaptive immunity. Direct interactions with autoreactive T-cells can activate mast cells, leading to cytokine production, including tumor necrosis factor α (TNF-α), IL6, and IL1β, following mast cell activation [33]. In conditions characterized by inflammation, such as in patients with defective spermatogenesis, varicocele, infertility, or autoimmune orchitis, there is an increase in the number of mast cells [34,35].

T-lymphocytes, following exposure to environmental signals, undergo commitment to either regulatory or effector lineages, each exhibiting distinct functions that contribute to either the establishment of immunologic tolerance or inflammation [36]. Several T-cell subtypes have been identified within mammalian testes. Although they represent a small portion of the testicular immune cells, approximately 10–20% of the total immune cells in the adult rat testes under normal physiological conditions [37], these cells potentially play a critical role in preserving immune tolerance and responding to pathogenic challenges during testicular infection and inflammation. Collectively, the testicular T-cell population includes regulatory T (T_reg_)-cells, helper T (T_h_)-cells, cytotoxic T (T_c_)-cells, natural killer (NK) T-cells, and γδ T-cells [38]. Regulatory T-cells, primarily found in the draining lymph nodes of the testes, play an important role in maintaining testicular immune privilege [39], and their presence within the normal testis is well-established [40]. Under normal physiological conditions, T_reg_ cells secrete inhibitory cytokines, including IL-10, IL-35, and transforming growth factor β (TGF-β), and through several immunosuppressive mechanisms, they control effector T-cells and prevent excessive immune response and autoimmunity [41]. Under pathological conditions, the expanding effector T-cells may overpower the suppressive mechanisms of T_reg_ cells, provoking an autoimmune response and leading to impaired spermatogenesis, autoimmune orchitis, and/or azoospermia [42]. Notably, T_reg_ cells are ineffective in preventing an attack on germ cells, possibly due to the cytokines within the inflammatory milieu compromising their efficacy at sites of inflammation [43]. Helper T-cells are classified into subgroups based on their secretion pattern, serving a distinct immunological role. For instance, T_h_1 cells secrete cytokine interferon-γ, IL-2, and TNF-α, contributing to antiviral and antibacterial immunity. T_h_2 cells secrete IL-4, IL-5, and IL-13 and are involved in combating extracellular pathogens, and T_h_17 cells play a role in antifungal defense and bacterial infection through the secretion of inflammation cytokines IL-7A, IL-17F, and IL-22. Notably, the T_h_ cell dynamic in the testes is crucial for maintaining homeostasis, and imbalances may lead to infection or chronic orchitis [44]. Cytotoxic T-cells are the most prominent T-cells in the testicular interstitium [38]. Their function involves the secretion of cytotoxic molecules, antiviral cytokines, and TNF-β to eliminate cells infected by pathogens, damaged cells, and malignant tumor cells. Moreover, their functional association with the quantity of testicular macrophages suggests a role in graft survival [45]. Natural killer (NK) T-cells possess immunoregulatory properties. They enhance immune responses to tumors and infectious diseases while suppressing cell-mediated immune reactions linked to autoimmune diseases and allograft rejection. γδ T-cells exhibit features of both innate and adaptive immunity, bridging these responses and contributing to antimicrobial and antitumor immunosurveillance, and they may amplify adaptive immune responses [38].

Given the intimate role of immune cells and cytokines in immune tolerance, the inflammation and dysregulation of the immune system might impact the immune-privileged status of the testis and spermatogenesis. Therefore, we hypothesize that an alteration of circulating immune cells and a systemic elevation of cytokine levels by immunotherapy may directly impact male fertility.

### 3.3. Testicular Cells

Different types of testicular cells actively secrete immunomodulatory molecules essential for establishing and maintaining the immune-privileged status of the testis. Sertoli cells release anti-inflammatory cytokines, activin A, TGF-β, and galectin-1, which inhibit the expression of proinflammatory cytokines in debritic cells and macrophages, contributing to immune-response suppression [46]. Tissue transplantation studies have further validated their immune protective role, leading to prolonged graft survival when co-transplanted with Sertoli cells [47]. Furthermore, since damaged germ cells can induce inflammatory responses in the testis [48], the timely removal of apoptotic germ cells and residual bodies by Sertoli cells is significant in preventing autoimmune responses [22]. Leydig cells also modulate immune responses. These cells secrete testosterone, known for its immunosuppressive properties by acting on Sertoli cells, and macrophage migration inhibitory factor, which inhibits the cytotoxic activities of T-cells [49]. Furthermore, peritubular cells contribute to testicular immune tolerance by expressing activin A and toll-like receptor (TLR) [50]. Also, male germ cells have been identified as sources of various cytokines, including IL-1a and TNF-α, suggesting a potential role in regulating the immune response [51]. Additionally, spermatogonia release soluble programmed death-ligand 1 (sPD-L1) to induce T-cell apoptosis [21].

## 4. Endocrinological and Testicular Changes during Development

From birth, through puberty, and to adulthood, the secretion of hormones in males changes drastically and, in response to the changing endocrine conditions, the testis undergoes distinctive cellular modifications [52]. Immediately after birth, the levels of gonadotrophins and testosterone increase (mini-puberty) before entering a “quiescent” period, marked by a decline in hormone levels. Specifically, follicle-stimulating hormone (FSH) and luteinizing hormone (LH) peak at 4–10 weeks post-natal, before reaching their lowest levels at around 6 months. Similarly, testosterone production from Leydig cells reaches its peak at approximately the third month and declines to prepubertal levels at 6–9 months. The absence of androgen receptor (AR) expression in immature Sertoli cells during this period delays further spermatogenesis, while anti-Müllerian hormone (AMH) production remains high [53]. In the testis, shortly after birth, gonocytes undergo proliferation until 6 months of age and differentiate into A_dark_ spermatogonia, which are considered the “true” spermatogonial stem cells (SSCs). During prepubertal life until the onset of spermatogenesis at puberty, spermatogonia constitute the only germ cell population. In the first year of life, the prepubertal testis harbors immature proliferative Sertoli cells, with fetal Leydig cells persisting until 6 months post-natally, subsequently being replaced by adult Leydig cell precursors (immature Leydig cells) [53,54].

Around puberty, pulsatile gonadotrophin-releasing hormone (GnRH) secretion initiates a progressive surge in gonadotrophin release. FSH promotes the proliferation of immature Sertoli cells, and LH induces the maturation of Leydig cells into adult Leydig cells, resuming testosterone production [53,55]. Elevated testosterone levels stimulate the maturation of Sertoli cells, which now express AR and are unable to undergo further mitotic division, and inhibit AMH expression [55]. During this period, the expansion of the lumen and the development of a layer of mature peritubular myoid cells separating seminiferous tubules from the interstitial compartment take place. Notably, in the developing human testis, the junctional specializations between Sertoli cells, building the BTB, are absent until approximately 8 years of age. However, these junctional structures begin to assemble in the early phase of puberty, typically occurring between 11 and 13 years of age [56]. Ultimately, germ cells can enter meiosis, completing their differentiation into haploid spermatozoa, with a directional progression from the basement membrane towards the lumen [52].

## 5. PD-1/PD-L1 and CTLA-4 Expression in the Testis

The exact function of the PD-1/PD-L1 pathway in testicular processes is not yet fully established. PD-1/PD-L1 serves as an additional T-cell tolerance system, with PD-L1 inhibiting T-cell activation through its interaction with PD-1 [57]. Notably, PD-L1 plays a role in the survival of islet allografts, suggesting that the PD-1/PD-L1 system serves as a mechanism underlying testicular immune privilege [58].

A few studies have attempted to evaluate the expression and localization of PD-1 and PD-L1 in the testis, with controversial results. Initially, PD-L1 was found to have an inducible expression on Sertoli cells but was consistently expressed on peritubular cells in the testes of mice [59]. However, an allograft study revealed that spermatocytes and spermatids were the primary cell types expressing PD-L1 in the seminiferous tubules [58]. A more recent study reported the detection of both PD-1 and PD-L1 in the testicular tissue of adult mice, as well as their age-related expression and localization [60]. PD-1 was mainly localized to advanced germ cells (elongating spermatids and spermatozoa), suggesting a potential role in spermiogenesis, with occasional PD-1 staining observed in the interstitial area. Since PD-1 was initially found in T-cells and is associated with programmed cell death, the researchers postulated the hypothesis that the expression of PD-1 in germ cells might be implicated in programmed cell death. Additionally, PD-L1 was expressed in the nucleus of Sertoli cells regardless of the testis developmental stage, allowing for the secretion of sPD-L1 into the testicular interstitial space, suggesting a role in the regulation of testicular immune privilege. Studies on normal human testis reported little or no PD-L1 expression [61,62] and no PD-1 expression [62,63]. The different results obtained from the aforementioned studies may be due to the utilization of different antibodies, distinct mouse strains with different genetic backgrounds, or variations in the detection methodology [60]. Overall, the discrepancies in the expression patterns complicate the precise identification of the function of the PD-1/PD-L1 pathway in testicular processes.

A new role for PD-L1 in the testis was revealed when researchers generated PD-L1 transgenic mice to investigate the physiological function of PD-L1 and its mechanism of action in various diseases [64]. Overexpression of PD-L1 in the testis caused abnormal testicular shrinkage and infertility in mice that were associated with abnormalities in spermatogenesis, including malformation and sloughing during spermatid development and a disorganized and collapsed seminiferous epithelium. This spermatogenic failure was only observed when PD-L1 was simultaneously expressed on Sertoli cells and spermatogonia, not when it was expressed only on spermatogonia. Based on these results, the researchers hypothesized that PD-L1 might engage in a self-interacting binding with PD-L1 on Sertoli cells during the early stages of spermatogenesis, potentially leading to sperm cell sloughing, suggesting a regulatory role of PD-L1 in microtubule organization and cell adhesion function. Recently, Shinohara and colleagues demonstrated that the enhancement of PD-L1 expression in SSCs enabled allogeneic offspring production in mice [65]. PD-L1 expression was induced by activating the MAPK14-BCL6B pathway, which promotes self-renewal through the generation of reactive oxygen species. The overexpression of PD-L1 on SSCs altered their immunological properties, enabling them to overcome the allogeneic barrier and make allogeneic recipients into “surrogate fathers”.

As of our current understanding, no studies have been conducted to investigate the expression of CTLA-4 in the testis and its potential role in testicular functions.

## 6. Direct and Indirect Effects on Testicular Function

**Figure 1:**
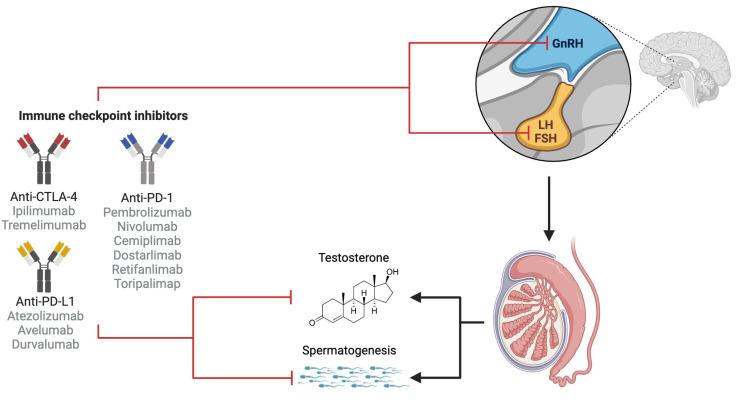
Potential effects of immune checkpoint inhibitors on the male reproductive system. Anti-PD-1, anti-PD-L1, and anti-CTLA-4 could directly impact male fertility by disrupting testosterone production and spermatogenesis. Additionally, their indirect effects could alter the hypothalamic–pituitary–gonadal axis, affecting the hormonal regulation of the male reproductive system.

The risk of gonadotoxicity associated with ICIs and the underlying mechanisms are currently not well-defined. It is hypothesized that there might be either a direct effect of ICIs on the testis and testosterone levels (primary hypogonadism) [66] or increased inflammation, or an indirect effect through endocrine dysfunction due to IrAEs, such as hypothyroidism or hypophysitis, resulting in decreased testosterone levels (secondary hypogonadism) [67] (Figure 1).

In the absence of preclinical studies, most data derive from small retrospective studies. The first retrospective study to investigate the impact of ICI therapy on testicular function and spermatogenesis included seven patients (age range: 23–78 years) treated with anti-CTLA-4 (ipilimumab) and anti-PD-1 (nivolumab) for over a month, at Johns Hopkins University Hospital, who ultimately succumbed to metastatic melanoma [68]. The post-mortem examination of testicular biopsies revealed impaired spermatogenesis in six patients, including focal spermatogenesis (n = 1), hypospermatogenesis (n = 2), and Sertoli cell-only syndrome (n = 3). No signs of increased peritubular hyalinization or fibrosis, nor Leydig cell abnormalities, were observed in any of the patients. This finding may indicate that ICI therapy could affect male fertility, an irAE that has previously been overlooked. However, due to the limited sample size, a direct correlation between ICI treatment and impaired spermatogenesis cannot be firmly established. The same group also reported a case of a normozoospermic 30-year-old patient treated for BRAF-negative stage IV metastatic melanoma, who developed azoospermia (Sertoli cell-only syndrome) two years after combined treatment with anti-PD1 and anti-CTLA4 (ipilimumab/nivolumab) [69]. The microscopic testicular sperm extraction performed five years after the patient’s ICI therapy also failed to retrieve any viable sperm. In a recent small cross-sectional study, including 25 men (age range: 26–59 years) undergoing ICI therapy for melanoma or cutaneous malignant tumors, the potential impact of these treatments on fertility was evaluated by semen and hormonal analysis [70]. Approximately 20 months post-treatment, all patients reported normal sexual function and most patients (18/22, 82%) had a normal semen analysis. However, three patients were diagnosed with azoospermia and one with oligoasthenoteratozoospermia. In three of the infertile patients, significant confounding factors (history of testis radiation, alcohol abuse, chemotherapy, bacterial orchitis) were identified, making the influence of ICI therapy on fertility unlikely in at least two cases. Interestingly, two patients developed autoimmune hypophysitis, and another patient developed autoimmune thyroiditis. The researchers concluded that one case of azoospermia, with the patient showing an asymptomatic, inflammatory infiltrate with neutrophil granulocytes, macrophages, and T-lymphocytes in the ejaculate, was likely ICI-related, and another case showed a significant worsening of seminal parameters. Although the majority of patients were not affected, a potential risk for an inflammatory loss of spermatogenesis seems possible.

There is limited clinical evidence suggesting that ICI therapy-induced primary hypogonadism from orchitis and secondary hypogonadism due to hypophysitis may pose a potential risk for male infertility [66,71]. Malfunctions of the BTB with damage to the germ epithelium, inflammation and impaired spermatogenesis might be induced by ICIs leading to autoimmune orchitis. Two reported cases involve patients with metastatic melanoma who encountered acute painful swelling of the testes. In one instance, a 54-year-old man experienced bilateral orchitis while undergoing ipilimumab-nivolumab (anti-PD-1/anti-CTLA-4) treatment. This was accompanied by abnormally low levels of testosterone and significantly high levels of LH, which is indicative of primary hypogonadism [72]. Hormonal levels spontaneously recovered, but since the patient did not consent to a semen analysis, the impact of this transient orchitis on spermatogenesis is unknown. The second case involved a patient who developed epididymo-orchitis while receiving pembrolizumab (anti-PD-1) treatment [73]. In this case, high-dose steroids were administered and there was a subsequent regression of symptoms, although hormone levels were not measured.

Additionally, endocrine autoimmune side effects may adversely impact fertility. Following an increasing trend since 2017, endocrine-related disorders are frequent irAEs of ICI therapy, involving disruptions in pituitary, thyroid, and adrenal functions, as well as diabetes [74,75,76]. Endocrine side effects of any grade are seen in up to 10% of patients receiving ICI monotherapy [77] or in up to 30% in the case of combined ICI therapy [78]. A retrospective single-center analysis of melanoma patients demonstrated that 11 out of 134 male patients (8%) developed hypophysitis following anti-CTLA-4 (ipilimumab) treatment within four months after the first dose. Even in the absence of hypophysitis, low testosterone levels were reported [74]. Interestingly, in cases of combined CTLA-4 and PD-1 blockade, the associated risk of hypophysitis, as well as thyroid dysfunction, was higher. Although patients received hormone replacement therapy, endogenous hormone secretion rarely recovered. Several other studies also reported similar incidences of hypophysitis after ICI therapy, with higher prevalence in males, and gonadotrophin deficiency [79,80]. In a recent analysis of VigiBase, the World Health Organization’s adverse drug reaction database, Bai and colleagues reported a higher and disproportionate risk of hypogonadism in men compared to women following treatment with ICIs [76]. Interestingly, the spectrum of endocrine-related disorders induced by various ICI therapies demonstrated notable distinctions. Anti-CTLA-4 monotherapy exhibited a higher association with hypophysitis (leading to hypopituitarism) and adrenal insufficiency, while anti-PD-1/PD-L1 monotherapy was found to be predominantly linked to thyroid dysfunction and type 1 diabetes mellitus. Additionally, combination therapy (anti-CTLA-4 plus anti-PD-1/PD-L1) covered almost all endocrine-related disorders and exhibited a stronger association compared to monotherapy, confirming previous studies on the increased risk for ICI-related endocrinopathies following combined treatment [76,78,81]. Of note, a previous study based on the French Pharmacovigilance database reported hypophysitis to occur with any type of currently available ICI, at any time of treatment, and regardless of the type of cancer [82]. Interestingly, hormonal function did not recover in any of the patients with gonadotrophic hormonal supplementation being suggested as part of their long-term onco-endocrinological care.

Treatment with anti-PD-1 and/or anti-CTLA-4 was also associated with low testosterone levels in 34 of 49 (69%) men with melanoma [83]. Furthermore, four patients developed hypophysitis and subsequent hypopituitarism while receiving ipilimumab. Individuals with stage 3 or 4 melanoma undergoing immunotherapy seemed to face an elevated risk of developing testosterone deficiency during their treatment.

In the preclinical setting, the only available data on the potential impact of ICIs on male fertility derive from studies performed by pharmaceutical companies. According to FDA reports, most of the clinically approved drugs do not have an effect on the male reproductive organs or fertility parameters in monkeys (Table 3). Only one drug (anti—PD-1/anti-LAG3) has been shown to cause inflammation (epididymis, seminal vesicles, and testes), while for the two anti-CTLA-4 drugs, such data are lacking. Unfortunately, access to more detailed data from these studies is not provided.

Given the recent approval of ICIs for pediatric patients, there is currently a lack of clinical evidence regarding their potential impact on the prepubertal testis. Additionally, there are no preclinical studies conducted. Moreover, only two (anti-PD-1: pembrolizumab and nivolumab) out of the six FDA-approved drugs for pediatric use have been tested on non-sexually matured monkeys with results reporting “no significant effects on the reproductive organs” (Table 3).

## 7. Discussion: Fertility Preservation before ICI Treatment and Future Perspectives

Infertility is a major side effect of cancer treatment [84]. With the development of more efficient drugs, patients have increased chances of survival. As a result, there is a growing population of adult, but also pediatric, cancer survivors who may face infertility. The impact of conventional cancer treatments, including radio- and chemotherapy, on the testis is well established. Several doses of irradiation and fractionation, as well as some chemotherapeutic drugs, especially alkylating and platinum-based agents, may cause infertility [85,86]. These treatments may affect both the adult and the prepubertal testis. Therefore, cryopreserving sperm before initiating such therapy can provide adult men with the opportunity to have biological children post-treatment. For prepubertal and early pubertal boys who cannot yet produce sperm, experimental testicular tissue banking before treatment is the sole option to safeguard their fertility [87,88]. While there are several indications for testicular tissue banking, ICI treatment and immunotherapy in general are not included due to the lack of evidence [86].

Although in the last decade a substantial number of clinical trials using ICIs have been conducted, the absence of data on fertility, and male fertility in particular, is staggering. None of the trials leading to the FDA approval of ICIs for various indications have included information on testicular function, sex hormone levels, or sexual health-related quality of life. At the same time, the use of ICIs continues to expand to various cancer types and younger ages of patients, which will result in more men and boys being exposed in the near future.

Despite the limited data currently available on the impact of ICIs on spermatogenesis and testicular function, the guidelines from the European Society for Medical Oncology and the European Society of Human Reproduction and Embryology emphasize the importance of fertility counseling for all patients [66]. A step further, the American Society of Clinical Oncology recommends sperm cryopreservation in males undergoing such treatments [89]. These recommendations reflect the cautious approach taken in light of the potential impact of ICIs on male fertility. Moreover, they highlight the need for comprehensive discussions regarding reproductive health in the context of oncology treatment, together with a patient-centered approach that considers individual circumstances and concerns.

Currently, there are no (pre)clinical data available on the potential impact of ICIs on the prepubertal testis. In contrast to previous knowledge [90], the prepubertal testis is potentially more susceptible to conventional oncological treatments compared to the adult testis due to the continuous turnover of early germ cells [91,92]. As reviewed earlier, limited data suggest the expression of PD-1 and PD-L1 on somatic and germ cells in the mouse testis. However, their function and the pathways they are involved in, both in mouse and human testes, are not known. Also, the expression of CTLA-4 has not yet been studied. In combination with the absence of the completely formed BTB before early puberty, this may render testicular cells vulnerable to treatments with anti-PD-1, anti-PD-L1, and anti-CTLA-4, resulting in a potential disruption of spermatogenesis. Additionally, from early puberty onwards, the activation of the hypothalamic–pituitary–gonadal axis and the initiation of spermatogenesis in response to gonadotrophin stimulation take place. Therefore, treatment with ICIs may affect sex hormonal regulation and testosterone production similarly to adult patients. Consequently, although there are cases reported where sperm collection and cryopreservation have been successfully performed for boys as young as 12 years old [93,94], it is possible that immature testicular tissue banking will be the only option for fertility preservation. In the case of pediatric patients, the identification of reproductive toxicity associated with new treatments may extend over a period of up to two decades or even longer, given their young age. Therefore, it becomes imperative to conduct preclinical studies employing animal models and/or human immature testicular biopsies to gain insights into the potential reproductive effects of these treatments. Such investigations are crucial for understanding the long-term impact on reproductive health in pediatric populations and the necessity for fertility preservation before the initiation of the treatment.

## 8. Conclusions

In summary, while ICIs have revolutionized cancer treatment, their potential impact on male fertility presents a growing concern. Although some mechanisms are not fully understood, it is clear that ICIs can cause reproductive dysfunction. The observed adverse effects, encompassing impaired spermatogenesis and endocrine-related disorders, highlight the importance of considering fertility-preservation strategies. Further research efforts, however, are essential to fully understand the mechanisms and potential off-target and long-term effects of ICIs on male reproductive health. Nevertheless, clinicians should be vigilant, and discussions about the potential reproductive risks and fertility preservation with the adult patients, or their primary caregivers in the case of pediatric patients, should be integrated into the comprehensive care.

## Figures and Tables

**Table 1:** FDA-approved immune checkpoint inhibitors and their indications for adult patients.

Drug (Brand Name)	First Approval Date	Drug Target	Indications
Ipilimumab (Yervoy)	25 March 2011	CTLA-4	Melanoma, renal cell carcinoma, colorectal cancer, hepatocellular carcinoma, and non-small cell lung cancer
Pembrolizumab (Keytruda)	4 September 2014	PD-1	Melanoma, non-small cell lung cancer, head and neck squamous cell cancer, classical Hodgkin lymphoma, primary mediastinal large B-cell lymphoma, urothelial carcinoma, microsatellite instability-high or mismatch repair deficient cancer, microsatellite instability-high or mismatch repair deficient colorectal cancer, gastric cancer, esophageal cancer, cervical cancer, hepatocellular carcinoma, Merkel cell carcinoma, renal cell carcinoma, endometrial carcinoma, tumor mutational burden-high cancer, cutaneous squamous cell carcinoma, and triple-negative breast cancer
Nivolumab (Opdivo)	22 December 2014	PD-1	Melanoma, non-small cell lung cancer, malignant pleural mesothelioma, renal cell carcinoma, classical Hodgkin lymphoma, squamous cell carcinoma of the head and neck, urothelial carcinoma, colorectal cancer, hepatocellular carcinoma, esophageal cancer, gastric cancer, gastroesophageal junction cancer, and esophageal adenocarcinoma
Atezolizumab (Tecentriq)	18 May 2016	PD-L1	Urothelial carcinoma, non-small cell lung cancer, small cell lung cancer, hepatocellular carcinoma, and melanoma
Avelumab (Bavencio)	23 March 2017	PD-L1	Merkel cell carcinoma, urothelial carcinoma, and renal cell carcinoma
Durvalumab (Imfinzi)	1 May 2017	PD-L1	Non-small cell lung cancer, small cell lung cancer, biliary tract cancer, and hepatocellular carcinoma
Cemiplimab (Libtayo)	28 September 2018	PD-1	Cutaneous squamous cell carcinoma, basal cell carcinoma, and non-small cell lung cancer
Dostarlimab (Jemperli)	22 April 2021	PD-1	Endometrial cancer
Nivolumab and Relatlimab (Opdualag)	18 March 2022	PD-1, LAG-3	Melanoma
Tremelimumab (Imjudo)	21 October 2022	CTLA-4	Hepatocellular carcinoma
Retifanlimab (Zynyz)	22 March 2023	PD-1	Merkel cell carcinoma
Toripalimab (Loqtorzi)	27 October 2023	PD-1	Nasopharyngeal carcinoma

PD-1: programmed death protein 1, PD-L1: programmed death-ligand 1, LAG-3: lymphocyte activation gene-3, CTLA-4: cytotoxic T-lymphocyte-associated protein 4.

**Table 2:** FDA-approved immune checkpoint inhibitors and their indications for pediatric patients.

Drug (Brand Name)	First Approval Date	Drug Target	Indications	Age Range
Avelumab (Bavencio)	23 March 2017	PD-L1	Merkel cell carcinoma	12 years and older
Pembrolizumab (Keytruda)	23 May 2017	PD-1	Melanoma, classical Hodgkin lymphoma, primary mediastinal B-cell lymphoma, microsatellite instability-high or mismatch repair deficient cancer, Merkel cell carcinoma, and tumor mutational burden-high cancer	12 years and older
Ipilimumab (Yervoy)	10 July 2018	CTLA-4	Melanoma and colorectal cancer	12 years and older
Nivolumab (Opdivo)	10 July 2018	PD-1	Melanoma and colorectal cancer	12 years and older
Nivolumab and Relatlimab (Opdualag)	18 March 2022	PD-1, LAG-3	Melanoma	12 years and older
Atezolizumab (Tecentriq)	9 December 2022	PD-L1	Alveolar soft part sarcoma	2 years and older

PD-1: programmed death protein 1, PD-L1: programmed death-ligand 1, LAG-3: lymphocyte activation gene-3, CTLA-4: cytotoxic T-lymphocyte-associated protein 4.

**Table 3:** FDA evidence.

Drug (Brand Name)	Drug Target	Reproductive Findings
Ipilimumab (Yervoy)	CTLA-4	No fertility studies performed
Pembrolizumab (Keytruda)	PD-1	No notable effects in male reproductive organs in 1- and 6-month repeat-dose toxicology studies on monkeys; however, most animals in these studies were not sexually mature
Nivolumab (Opdivo)	PD-1	No significant effects on male reproductive organs in 1- and 3-month toxicology studies on monkeys; however, most animals in these studies were not sexually mature
Atezolizumab (Tecentriq)	PD-L1	No notable effects in male reproductive organs in a 26-week repeat-dose toxicity study in monkeys
Avelumab (Bavencio)	PD-L1	No notable effects in male reproductive organs in a 3-month repeat-dose toxicity study in monkeys
Durvalumab (Imfinzi)	PD-L1	No notable effects in male reproductive organs in 3-month repeat-dose toxicology studies on sexually mature monkeys
Cemiplimab (Libtayo)	PD-1	No effects on fertility parameters (semen analysis or testicular measurements) or in male reproductive organs in a 3-month repeat-dose toxicology study in sexually mature monkeys
Dostarlimab (Jemperli)	PD-1	No significant effects on male reproductive organs in 1- and 3-month toxicology studies on monkeys; however, most animals in these studies were not sexually mature
Nivolumab and Relatlimab (Opdualag)	PD-1, LAG-3	Inflammation within the reproductive tract (epididymis, seminal vesicles, and testes) was observed in a 1-month study in monkeys
Tremelimumab (Imjudo)	CTLA-4	No fertility studies performed
Retifanlimab (Zynyz)	PD-1	No significant effects on male reproductive organs in 1- and 3-month toxicology studies on monkeys; however, most animals in these studies were not sexually mature
Toripalimab (Loqtorzi)	PD-1	No notable effects in male reproductive organs in 4- and 26-week repeat-dose toxicology studies in sexually mature monkeys

PD-1: programmed death protein 1, PD-L1: programmed death-ligand 1, LAG-3: lymphocyte activation gene-3, CTLA-4: cytotoxic T-lymphocyte-associated protein 4.

## References

[1] Zhang H., Chen J. (2018). Current status and future directions of cancer immunotherapy. J. Canc..

[2] Smyth M.J., Teng M.W. (2018). 2018 Nobel Prize in physiology or medicine. CTI.

[3] Postow M.A., Sidlow R., Hellmann M.D. (2018). Immune-related adverse events associated with immune checkpoint blockade. N. Engl. J. Med..

[4] Mulder R.L., Font-Gonzalez A., Green D.M., Loeffen E.A.H., Hudson M.M., Loonen J., Yu R., Ginsberg J.P., Mitchell R.T., Byrne J. (2021). Fertility preservation for male patients with childhood, adolescent, and young adult cancer: Recommendations from the PanCareLIFE Consortium and the International Late Effects of Childhood Cancer Guideline Harmonization Group. Lancet Oncol..

[5] Pardoll D.M. (2012). The blockade of immune checkpoints in cancer immunotherapy. Nat. Rev. Cancer.

[6] Brunet J.F., Denizot F., Luciani M.F., Roux-Dosseto M., Suzan M., Mattei M.G., Golstein P. (1987). A new member of the immunoglobulin superfamily—CTLA-4. Nature.

[7] Zeng J., See A.P., Phallen J., Jackson C.M., Belcaid Z., Ruzevick J., Durham N., Meyer C., Harris T.J., Albesiano E. (2013). Anti-PD-1 blockade and stereotactic radiation produce long-term survival in mice with intracranial gliomas. Int. J. Radiat. Oncol. Biol. Phys..

[8] Sharpe A., Pauken K. (2018). The diverse functions of the PD1 inhibitory pathway. Nat. Rev. Immunol..

[9] Webb E.S., Liu P., Baleeiro R., Lemoine N.R., Yuan M., Wang Y. (2018). Immune checkpoint inhibitors in cancer therapy. J. Biomed. Res..

[10] Wei S.C., Duffy C.R., Allison J.P. (2018). Fundamental mechanisms of immune checkpoint blockade therapy. Cancer Discov..

[11] Pawłowska A., Suszczyk D., Okła K., Barczynski B., Kotarski J., Wertel I. (2019). Immunotherapies based on PD-1/PD-L1 pathway inhibitors in ovarian cancer treatment. Clin. Exp. Immunol..

[12] Egen J., Kuhns M., Allison J. (2002). CTLA-4: New insights into its biological function and use in tumor immunotherapy. Nat. Immunol..

[13] Hoos A. (2016). Development of immuno-oncology drugs—From CTLA4 to PD1 to the next generations. Nat. Rev. Drug. Discov..

[14] Sharma P., Goswami S., Raychaudhuri D., Siddiqui B.A., Singh P., Nagarajan A., Liu J., Subudhi S.K., Poon C., Gant K.L. (2023). Immune checkpoint therapy-current perspectives and future directions. Cell.

[15] ClinicalTrials.gov. https://clinicaltrials.gov/.

[16] Vaddepally R.K., Kharel P., Pandey R., Garje R., Chandra A.B. (2020). Review of indications of FDA-approved immune checkpoint inhibitors per NCCN guidelines with the level of evidence. Cancers.

[17] Tanda E.T., Croce E., Spagnolo F., Zullo L., Spinaci S., Genova C., Rossi G. (2021). Immunotherapy in adolescents and young adults: What remains in cancer survivors?. Front. Oncol..

[18] Tan S., Day D., Nicholls S.J., Segelov E. (2022). Immune checkpoint inhibitor therapy in oncology: Current uses and future directions. Cardio Oncol..

[19] Drugs@FDA: FDA-Approved Drugs. https://www.accessdata.fda.gov/scripts/cder/daf/index.cfm.

[20] Pediatric Oncology Drug Approvals. https://www.fda.gov/about-fda/oncology-center-excellence/pediatric-oncology-drug-approvals.

[21] Chen Q., Deng T., Han D. (2016). Testicular immunoregulation and spermatogenesis. Semin. Cell Dev. Biol..

[22] Zhao S., Zhu W., Xue S., Han D. (2014). Testicular defense systems: Immune privilege and innate immunity. Cell. Mol. Immunol..

[23] Khambata K., Modi D.N., Gupta S.K. (2021). Immunoregulation in the testis and its implication in fertility and infections. Explor. Immunol..

[24] Cheng C.Y., Mruk D.D. (2012). The blood–testis barrier and its implications for male contraception. Pharmacol. Rev..

[25] Fijak M., Bhushan S., Meinhardt A. (2011). Immunoprivileged sites: The testis. Methods Mol. Biol..

[26] Bhushan S., Theas M.S., Guazzone V.A., Jacobo P., Wang M., Fijak M., Meinhardt A., Lustig L. (2020). Immune cell subtypes and their function in the testis. Front. Immunol..

[27] Hedger M.P. (2002). Macrophages and the immune responsiveness of the testis. J. Reprod. Immunol..

[28] Wang M., Fijak M., Hossain H., Markmann M., Nüsing R.M., Lochnit G., Hartmann M.F., Wudy S.A., Zhang L., Gu H. (2017). Characterization of the micro-environment of the testis that shapes the phenotype and function of testicular macrophages. J. Immunol..

[29] Winnall W.R., Muir J.A., Hedger M.P. (2011). Rat resident testicular macrophages have an alternatively activated phenotype and constitutively produce interleukin-10 in vitro. J. Leukoc. Biol..

[30] Mossadegh-Keller N., Sieweke M.H. (2018). Testicular macrophages: Guardians of fertility. Cell. Immunol..

[31] Rival C., Guazzone V.A., von Wulffen W., Hackstein H., Schneider E., Lustig L., Meinhardt A., Fijak M. (2007). Expression of co-stimulatory molecules, chemokine receptors and proinflammatory cytokines in dendritic cells from normal and chronically inflamed rat testis. Mol. Hum. Reprod..

[32] Duan Y.G., Yu C.F., Novak N., Bieber T., Zhu C.H., Schuppe H.C., Haidl G., Allam J.P. (2011). Immunodeviation towards a Th17 immune response associated with testicular damage in azoospermic men. Int. J. Androl..

[33] Haidl G., Duan Y.G., Chen S.J., Kohn F.M., Schuppe H.C., Allam J.P. (2011). The role of mast cells in male infertility. Expert Rev. Clin. Immunol..

[34] Mayerhofer A., Walenta L., Mayer C., Eubler K., Welter H. (2018). Human testicular peritubular cells, mast cells and testicular inflammation. Andrologia.

[35] Moreno D., Sobarzo C.M., Lustig L., Rodriguez Pena M.G., Guazzone V.A. (2020). Effect of ketotifen fumarate on experimental autoimmune orchitis and torsion of the spermatic cord. Asian J. Androl..

[36] Khan U., Ghazanfar H. (2018). T lymphocytes and autoimmunity. Int. Rev. Cell. Mol. Biol..

[37] Hedger M.P. (1997). Testicular leukocytes: What are they doing?. Rev. Reprod..

[38] Gong J., Zeng Q., Yu D., Duan Y.G. (2020). T lymphocytes and testicular immunity: A new insight into immune regulation in testes. Int. J. Mol. Sci..

[39] Garza K.M., Agersborg S.S., Baker E., Tung K.S. (2000). Persistence of physiological self-antigen is required for the regulation of self-tolerance. J. Immunol..

[40] Wheeler K., Tardif S., Rival C., Luu B., Bui E., Del Rio R., Teuscher C., Sparwasser T., Hardy D., Tung K.S. (2011). Regulatory T cells control tolerogenic versus autoimmune response to sperm in vasectomy. Proc. Natl. Acad. Sci. USA.

[41] Nasr I.W., Wang Y., Gao G., Deng S., Diggs L., Rothstein D.M., Tellides G., Lakkis F.G., Dai Z. (2005). Testicular immune privilege promotes transplantation tolerance by altering the balance between memory and regulatory T cells. J. Immunol..

[42] Jacobo P., Guazzone V.A., Jarazo-Dietrich S., Theas M.S., Lustig L. (2009). Differential changes in CD4+ and CD8+ effector and regulatory T lymphocyte subsets in the testis of rats undergoing autoimmune orchitis. J. Reprod. Immunol..

[43] Jacobo P. (2018). The role of regulatory T Cells in autoimmune orchitis. Andrologia.

[44] Jacobo P., Perez C.V., Theas M.S., Guazzone V.A., Lustig L. (2011). CD4+ and CD8+ T cells producing Th1 and Th17 cytokines are involved in the pathogenesis of autoimmune orchitis. Reproduction.

[45] Hedger M.P., Meinhardt A. (2000). Local regulation of T cell numbers and lymphocyte-inhibiting activity in the interstitial tissue of the adult rat testis. J. Reprod. Immunol..

[46] Hedger M.P., Winnall W.R. (2012). Regulation of activin and inhibin in the adult testis and the evidence for functional roles in spermatogenesis and immunoregulation. Mol. Cell. Endocrinol..

[47] Suarez-Pinzon W., Korbutt G.S., Power R., Hooton J., Rajotte R.V., Rabinovitch A. (2000). Testicular Sertoli cells protect islet beta-cells from autoimmune destruction in NOD mice by a transforming growth factor-beta1-dependent mechanism. Diabetes.

[48] Zhang X., Wang T., Deng T., Xiong W., Lui P., Li N., Chen Y., Han D. (2013). Damaged spermatogenic cells induce inflammatory gene expression in mouse Sertoli cells through the activation of Toll-like receptors 2 and 4. Mol. Cell. Endocrinol..

[49] Fijak M., Schneider E., Klug J., Bhushan S., Hackstein H., Schuler G., Wygrecka M., Gromoll J., Meinhardt A. (2011). Testosterone replacement effectively inhibits the development of experimental autoimmune orchitis in rats: Evidence for a direct role of testosterone on regulatory T cell expansion. J. Immunol..

[50] Mayer C., Adam M., Glashauser L., Dietrich K., Schwarzer J.U., Köhn F.M., Strauss L., Welter H., Poutanen M., Mayerhofer A. (2016). Sterile inflammation as a factor in human male infertility: Involvement of Toll-like receptor 2, biglycan and peritubular cells. Sci. Rep..

[51] Haugen T.B., Landmark B.F., Josefsen G.M., Hansson V., Hogset A. (1994). The mature form of interleukin-1 alpha is constitutively expressed in immature male germ cells from rat. Mol. Cell. Endocrinol..

[52] Rey R.A. (2014). Mini-puberty and true puberty: Differences in testicular function. Ann. Endocrinol..

[53] Mitchell R.T., O’Hara L., Smith L.B., Oatley J.M., Griswold M.D. (2017). Gonadotropin and steroid hormone control of spermatogonial differentiation. The Biology of Mammalian Spermatogonia.

[54] Hermann B.P., Sukhwani M., Hansel M.C., Orwig K.E. (2010). Spermatogonial stem cells in higher primates: Are there differences from those in rodents?. Reproduction.

[55] Sharpe R.M., McKinnell C., Kivlin C., Fisher J.S. (2003). Proliferation and functional maturation of Sertoli cells, and their relevance to disorders of testis function in adulthood. Reproduction.

[56] de Kretser D.M., Loveland K., O’Bryan M., Jameson J.L., De Groot L.J., de Kretser D.D., Giudice L.C., Grossman A.B., Melmed S., Potts J.T., Weir G.C. (2016). Spermatogenesis. Endocrinology: Adult and Pediatric.

[57] Keir M.E., Butte M.J., Freeman G.J., Sharpe A.H. (2008). PD-1 and its ligands in tolerance and immunity. Annu. Rev. Immunol..

[58] Cheng X., Dai H., Wan N., Moore Y., Vankayalapati R., Dai Z. (2009). Interaction of programmed death-1 and programmed death-1 ligand-1 contributes to testicular immune privilege. Transplantation.

[59] Dal Secco V., Riccioli A., Padula F., Ziparo E., Filippini A. (2008). Mouse Sertoli cells display phenotypical and functional traits of antigen-presenting cells in response to interferon gamma. Biol. Reprod..

[60] Wang L.L., Li Z.H., Duan Y.G., Yuan S.Q., Mor G., Liao A.H. (2019). Identification of programmed cell death 1 and its ligand in the testicular tissue of mice. Am. J. Reprod. Immunol..

[61] Fankhauser C.D., Honecker F., Beyer J., Bode P.K. (2015). Emerging therapeutic targets for male germ cell tumors. Curr. Oncol. Rep..

[62] Cierna Z., Mego M., Miskovska V., Machalekova K., Chovanec M., Svetlovska D., Hainova K., Rejlekova K., Macak D., Spanik S. (2016). Prognostic value of programmed-death-1 receptor (PD-1) and its ligand 1 (PD-L1) in testicular germ cell tumors. Ann. Oncol..

[63] Jennewein L., Bartsch G., Gust K., Kvasnicka H.M., Haferkamp A., Blaheta R., Mittelbronn M., Harter P.N., Mani J. (2018). Increased tumor vascularization is associated with the amount of immune competent PD-1 positive cells in testicular germ cell tumors. Oncol. Lett..

[64] Fang L., Feng R., Liang W., Liu F.F., Bian G.L., Yu C., Guo H., Cao Y., Liu M., Zuo J. (2022). Overexpression of PD-L1 causes germ cells to slough from mouse seminiferous tubules via the PD-L1/PD-L1 interaction. J. Cell. Mol. Med..

[65] Shinohara T., Yamamoto T., Morimoto H., Shiromoto Y., Kanatsu-Shinohara M. (2023). Allogeneic offspring produced by induction of PD-L1 in spermatogonial stem cells via self-renewal stimulation. Stem Cell Rep..

[66] Garutti M., Lambertini M., Puglisi F. (2021). Checkpoint inhibitors, fertility, pregnancy, and sexual life: A systematic review. ESMO Open.

[67] Santaballa A., Márquez-Vega C., Rodríguez-Lescure Á., Rovirosa Á., Vázquez L., Zeberio-Etxetxipia I., Andrés M., Bassas L., Ceballos-Garcia E., Domingo J. (2022). Multidisciplinary consensus on the criteria for fertility preservation in cancer patients. Clin. Transl. Oncol..

[68] Scovell J.M., Benz K., Samarska I., Kohn T.P., Hooper J.E., Matoso A., Herati A.S. (2020). Association of impaired spermatogenesis with the use of immune checkpoint inhibitors in patients with metastatic melanoma. JAMA Oncol..

[69] Rabinowitz M.J., Kohn T.P., Peña V.N., Samarska I.V., Matoso A., Herati A.S. (2020). Onset of azoospermia in man treated with ipilimumab/nivolumab for BRAF negative metastatic melanoma. Urol. Case Rep..

[70] Salzmann M., Tosev G., Heck M., Schadendorf D., Maatouk I., Enk A.H., Hartmann M., Hassel J.C. (2021). Male fertility during and after immune checkpoint inhibitor therapy: A cross-sectional pilot study. Eur. J. Cancer.

[71] Özdemir B.C. (2021). Immune checkpoint inhibitor-related hypogonadism and infertility: A neglected issue in immuno-oncology. J. ImmunoTher. Cancer.

[72] Brunet-Possenti F., Opsomer M.A., Gomez L., Ouzaid I., Descamps V. (2017). Immune checkpoint inhibitors-related orchitis. Ann. Oncol..

[73] Quach H.T., Robbins C.J., Balko J.M., Chiu C.Y., Miller S., Wilson M.R., Nelson G.E., Johnson D.B. (2019). Severe epididymo-orchitis and encephalitis complicating anti-PD-1 therapy. Oncologist.

[74] Ryder M., Callahan M., Postow M.A., Wolchok J., Fagin J.A. (2014). Endocrine-related adverse events following ipilimumab in patients with advanced melanoma: A comprehensive retrospective review from a single institution. Endocr. Relat. Cancer.

[75] Nogueira E., Newsom-Davis T., Morganstein D.L. (2019). Immunotherapy-induced endocrinopathies: Assessment, management, and monitoring. Ther. Adv. Endocrinol. Metab..

[76] Bai X., Lin X., Zheng K., Chen X., Wu X., Huang Y., Zhuang Y. (2020). Mapping endocrine toxicity spectrum of immune checkpoint inhibitors: A disproportionality analysis using the WHO adverse drug reaction database, VigiBase. Endocrine.

[77] Eigentler T.K., Hassel J.C., Berking C., Aberle J., Bachmann O., Grünwald V., Kähler K.C., Loquai C., Reinmuth N., Steins M. (2016). Diagnosis, monitoring and management of immune-related adverse drug reactions of anti-PD-1 antibody therapy. Cancer Treat. Rev..

[78] Hassel J.C., Heinzerling L., Aberle J., Bähr O., Eigentler T.K., Grimm M.O., Grünwald V., Leipe J., Reinmuth N., Tietze J.K. (2017). Combined immune checkpoint blockade (anti-PD-1/anti-CTLA-4): Evaluation and management of adverse drug reactions. Cancer Treat. Rev..

[79] Faje A.T., Sullivan R., Lawrence D., Tritos N.A., Fadden R., Klibanski A., Nachtigall L. (2014). Ipilimumab-induced hypophysitis: A detailed longitudinal analysis in a large cohort of patients with metastatic melanoma. J. Clin. Endocrinol. Metab..

[80] Albarel F., Gaudy C., Castinetti F., Carré T., Morange I., Conte-Devolx B., Grob J.J., Brue T. (2015). Long-Term follow-up of ipilimumab-induced hypophysitis, a common adverse event of the anti-CTLA-4 antibody in melanoma. Eur. J. Endocrinol..

[81] Barroso-Sousa R., Barry W.T., Garrido-Castro A.C., Hodi F.S., Min L., Krop I.E., Tolaney S.M. (2018). Incidence of endocrine dysfunction following the use of different immune checkpoint inhibitor regimens: A systematic review and meta-analysis. JAMA Oncol..

[82] Garon-Czmil J., Petitpain N., Rouby F., Sassier M., Babai S., Yéléhé-Okouma M., Weryha G., Klein M., Gillet P. (2019). mmune checkpoint inhibitors-induced hypophysitis: A retrospective analysis of the French Pharmacovigilance database. Sci. Rep..

[83] Peters M., Pearlman A., Terry W., Mott S.L., Monga V. (2021). Testosterone deficiency in men receiving immunotherapy for malignant melanoma. Oncotarget.

[84] Poorvu P.D., Frazier A.L., Feraco A.M., Manley P.E., Ginsburg E.S., Laufer M.R., LaCasce A.S., Diller L.R., Partridge A.H. (2019). Cancer treatment-related infertility: A critical review of the evidence. JNCI Cancer Spectr..

[85] Meistrich M.L. (2013). Effects of chemotherapy and radiotherapy on spermatogenesis in humans. Fertil. Steril..

[86] Delgouffe E., Braye A., Goossens E. (2022). Testicular tissue banking for fertility preservation in young boys: Which patients should be included?. Front. Endocrinol..

[87] Picton H.M., Wyns C., Anderson R.A., Goossens E., Jahnukainen K., Kliesch S., Mitchell R.T., Pennings G., Rives N., Tournaye H. (2015). A European perspective on testicular tissue cryopreservation for fertility preservation in prepubertal and adolescent boys. Hum. Reprod..

[88] Goossens E., Jahnukainen K., Mitchell R.T., van Pelt A., Pennings G., Rives N., Poels J., Wyns C., Lane S., Rodriguez-Wallberg K.A. (2020). Fertility preservation in boys: Recent developments and new insights. Hum. Reprod. Open.

[89] Cosci I., Grande G., Di Nisio A., Rocca M.S., Del Fiore P., Benna C., Mocellin S., Ferlin A. (2022). Cutaneous Melanoma and hormones: Focus on sex differences and the testis. Int. J. Mol. Sci..

[90] Rivkees S.A., Crawford J.D. (1988). The relationship of gonadal activity and chemotherapy-induced gonadal damage. JAMA.

[91] de Rooij D.G., Creemers L.B., den Ouden K., Izadyar F., Rommerts F.F.G., Teerds K.J. (2002). Spermatogonial stem cell development. Testicular Tangrams.

[92] Jahnukainen K., Ehmcke J., Hou M., Schlatt S. (2011). Testicular function and fertility preservation in male cancer patients. Best Pract. Res. Clin. Endocrinol. Metab..

[93] Bahadur G., Ling K.L., Hart R., Ralph D., Wafa R., Ashraf A., Jaman N., Mahmud S., Oyede A.W. (2002). Semen quality and cryopreservation in adolescent cancer patients. Hum. Reprod..

[94] Hagenäs I., Jørgensen N., Rechnitzer C., Sommer P., Holm M., Schmiegelow K., Daugaard G., Jacobsen N., Juul A. (2010). Clinical and biochemical correlates of successful semen collection for cryopreservation from 12-18-year-old patients: A single-center study of 86 adolescents. Hum. Reprod..

